# Predicting Diabetic Foot Ulcer Outcomes: Machine Learning‐Based Refinement of IWGDF‐Approved Classifications for Outpatient Services

**DOI:** 10.1002/edm2.70193

**Published:** 2026-05-03

**Authors:** Farideh Mostafavi, Mohammad Reza Amini, Yadollah Mehrabi, Melina Rezvani, Mehrangiz Toutounchi, Seyed Saeed Hashemi Nazari

**Affiliations:** ^1^ Student Research Committee, School of Public Health and Safety Shahid Beheshti University of Medical Sciences Tehran Iran; ^2^ Department of Epidemiology, School of Public Health and Safety Shahid Beheshti University of Medical Sciences Tehran Iran; ^3^ Diabetes Research Center, Endocrinology and Metabolism Clinical Sciences Institute Tehran University of Medical Sciences Tehran Iran; ^4^ Department of Photo Healing and Regeneration Medical Laser Research Center, Yara Institute, ACECR Tehran Iran

**Keywords:** diabetic foot, foot ulcer/ulceration, machine learning, ulcer classification, validation

## Abstract

**Introduction:**

This study aimed to validate and compare six IWGDF‐approved classification systems for predicting poor prognostic outcomes in diabetic foot ulcers (DFUs), using the same sample in Iran. We also proposed modifications to enhance the performance and feasibility of these tools, in outpatient setting.

**Methods:**

A prospective cohort study was conducted involving 616 DFUs from 400 patients over a six‐month period. We assessed the performance of six wound classification systems: Wagner, UTWCS, PEDIS/IDSA, SINBAD, WIFI, and DiaFORA. We adjusted for the key variables associated with poor outcomes that could affect the performance of these systems, employing ten machine learning techniques along with the Least Absolute Shrinkage and Selection Operator (LASSO) and random forest methods for feature selection methods.

**Results:**

Our findings indicated that both the SINBAD and UTWCS systems exhibited comparable effectiveness in predicting outcomes, significantly surpassing other systems. Notably, modifications to the WIFI system—specifically, redefining the wound depth classification to a clearer category—yielded improved predictive capabilities, outperforming the existing systems like SINBAD and UTWCS in predicting poor prognostic outcomes in outpatient setting.

**Conclusion:**

SINBAD and UTWCS systems yield the best performance in our samples. Our proposed modification on the WIFI system can enhance its applicability for outpatient services according to reported performances.

## Graphical abstract

Six IWGDF‐approved diabetic foot ulcer classification systems were compared using machine‐learning models. SINBAD and UTWCS showed the strongest prediction of poor outcomes, while a modified WIFI system using simplified wound‐depth grading further improved predictive performance in outpatient care.

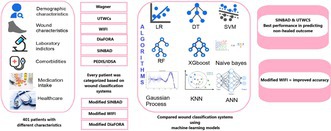

## 1 Introduction

Diabetic foot is a serious complication of diabetes with high morbidity and mortality rates [1], affecting 6.3% of the diabetic population, including 5.5% in Asia and Iran [2]. DFU is one of the most complex and heterogeneous complications of diabetes; however, its management is hindered by the lack of a universally accepted and clinically practical classification system [3]. Although many efforts have been made in recent years to develop international guidelines to provide standard care, data on outcomes and predictors in patients with DFU are limited [4]. Several classification systems have been devised to enable effective comparison of clinical outcomes; however, almost all of these systems have limitations and there is no universally agreed upon standard [5]. The International Working Group on Diabetic Foot (IWGDF) has systematically reviewed 28 wound classification tools across 149 articles. Most studies were assessed as having a high risk of bias, often reporting only unadjusted associations. Based on this review, the IWGDF identified six classification systems with relatively better performance in associational analyses of wound outcomes, while emphasising each system has its own limitations [6, 7]. These classification systems include Maggit‐Wagner, SINBAD (ulcer Site, Ischemia, Neuropathy, Bacterial infection, Area and Depth), University of Texas Wound Classification System (UTWCS), Infectious Diseases Society of America (IDSA) classification system, WIFI (Wound, Ischemia, Foot Infection) and DiaFORA (diabetic foot risk assessment tool) [6].

IWGDF recommends the use of the SINBAD system for communication between healthcare staff to manage ulcers and suggests the WIFI system if resources permit. However, they have stated that the conditions in which the classification system is supposed to be applied are not always clear, and current recommendations regarding the use of these systems are different based on societies, countries and the availability of procedures and specialised staff, so it is strongly recommended to conduct more validation studies [8, 9].

Current validation studies have primarily relied on generalised linear models, such as logistic regression and Cox proportional hazard models, to predict wound healing outcomes, which may not effectively capture the complex nature of DFUs [10, 11, 12]. In contrast, machine learning algorithms, a branch of artificial intelligence, have gained recognition for their diagnostic and prognostic capabilities, often outperforming traditional linear models in various applications [10]. Given the complexity and heterogeneity of wound characteristics and patient profiles, the integration of machine learning techniques is increasingly seen as a high priority in this field [8, 9]. Despite this, our knowledge indicates that previous validation studies of wound classification systems have not adequately employed advanced machine learning methods to evaluate the prognostic outcomes associated with each classification system. This gap underscores the need for innovative approaches that leverage machine learning to enhance the predictive power and clinical utility of existing DFU classification systems.

In this study, we initially validated the six IWGDF‐approved classification systems and assessed their predictive power for poor wound prognosis using machine learning algorithms in a prospective cohort study. Finally, to improve the performance of the existing classification systems or simplify their application, we proposed modifications to the well‐performing systems.

## 2 Material and Methods

### 2.1 Study Design and Participants

This prospective cohort study was conducted at the outpatient wound clinic of Tehran University of Medical Sciences. The cohort has been described previously in a study evaluating the prediction of amputation using diabetic foot ulcer (DFU) classification systems [13].

Briefly, patients with newly diagnosed DFUs were consecutively enrolled after providing written informed consent and followed for 6 months. Baseline demographic characteristics, diabetes‐related factors, comorbidities, laboratory markers, medication use and wound characteristics relevant to outpatient care were collected by trained physicians and nurses using a standardised checklist, as previously reported [13].

In the present study, six IWGDF‐endorsed wound classification systems (PEDIS, SINBAD, Wagner, UTWCS, WIFI and DiaFORA) were evaluated for their ability to predict non‐healing, representing a distinct clinical application from the prior amputation‐focused analysis.

### 2.2 Outcome Definition

The binary outcome was defined as follows:

1. Non‐healed (Class 1): failure to achieve complete wound healing within 6 months of baseline assessment, including patients who underwent minor or major amputation during follow‐up.
2. Healed (Class 0): complete wound healing, defined as full epithelialization of the skin

### 2.3 Statistical Analysis

Before doing any modifications on wound classification systems and compare their performance to predict DFU poor prognosis with original tools, in the first part, we determined the prediction power of the classification system. For this purpose, first associations between each classification system and non‐healing were assessed using univariate logistic regression. Multivariable prediction models were then developed using 10 machine learning algorithms. Each classification system was entered as the primary predictor and adjusted for relevant clinical variables.

The machine learning algorithms used in this study included random forest, decision trees, support vector machines (SVM), including radial basis function (RBF) and linear SVM, XGBoost, K‐nearest neighbours (KNN), Gaussian process, Naïve Bayes, logistic regression and deep neural networks. The data was divided into a training set (70%) and a test set (30%). Hyperparameter tuning was performed for deep neural networks, XGBoost, random forest and SVM algorithms, utilising fivefold cross‐validation. The architecture of the neural networks is detailed in the appendix file. The reported performance was based on test data.

To reduce overfitting, feature selection was performed using Least Absolute Shrinkage and Selection Operator (LASSO) and random forest methods. To prevent data leakage, the dataset was first split into training and test sets and then feature selection was applied separately on the train and test sets.

Algorithms included random forest, decision tree, XGBoost, SVM (linear and RBF), K‐nearest neighbours, Gaussian process, Naïve Bayes, logistic regression and deep neural networks. For each classification system, three feature sets were evaluated: Reduced model (random forest selection), reduced model (LASSO) and Full model (all predictors). The best‐performing method among the three feature sets was then used for the final comparison of wound classification systems.

Hyperparameter tuning using fivefold cross‐validation was performed for algorithms with substantial model complexity and tuning sensitivity, including deep neural networks, XGBoost, random forest and support vector machines with linear and radial basis function kernels. For other algorithms—logistic regression, Naïve Bayes, Gaussian process classifiers and decision trees—default or standard parameter settings were used, as these algorithms have limited tunable parameters and extensive tuning may lead to overfitting in a dataset of this size.

Since the outcome distribution was moderately imbalanced (64.6% healed and 35.4% non‐healed ulcers), several strategies were evaluated to address class imbalance during model development. We first explored data‐level approaches, including oversampling, under sampling and the Synthetic Minority Over‐sampling Technique (SMOTE). However, these methods did not improve model generalisation and, in some cases, reduced performance on the independent test set.

Consequently, we adopted algorithm‐level strategies to handle class imbalance. For deep neural network models, binary cross‐entropy with logits loss (BCEWithLogitsLoss) with class weighting was used to penalise misclassification of the minority (non‐healed) class. For other machine learning algorithms, class imbalance was addressed using class‐weighted loss functions or balanced class weights, where supported by the algorithm. To provide a complete evaluation of model performance under imbalance, we report per‐class metrics for the non‐healed class (positive class) including precision, recall (sensitivity) and F1‐score, in addition to specificity and AUC. These metrics are presented in Tables 1, 2, 3 and refer to Class 1 = non‐healed ulcers. This approach ensures that model performance is assessed fairly for the clinically important non‐healed outcome, rather than relying solely on accuracy, which can be misleading in imbalanced datasets.

**TABLE 1:** Performance of univariate model for predicting poor prognosis healing outcome during 6 months using logistic regression.

	Accuracy	F1 score	Precision	Recall	Specificity	AUC
Wagner	65.4	54.4	47.4	63.8	66.1	69.0
WIFI	63.7	57.0	46.2	74.1	58.7	73.0
UTWCS	67.0	56.3	49.4	65.5	67.8	71.0
SINBAD	67.0	55.6	49.3	63.8	68.6	68.0
IDSA	62.0	51.4	43.9	62.1	62.0	59.0
PEDIS	68.7	57.6	51.4	65.5	70.2	72.0
DiaFORA	68.7	50.0	51.8	48.3	78.1	67.0

Abbreviations: DiaFORA, Diabetic Foot Risk Assessment; IDSA, Infectious Diseases Society of America; PEDIS, perfusion, extent, depth/tissue loss, infection and sensation; SINBAD, site, ischemia, neuropathy, bacterial infection and depth; UTWCS, University of Texas Wound Classification System; WIfI, wound, ischemia and foot infection.

Because some patients contributed more than one ulcer, we accounted for within‐patient clustering during model development. To avoid data leakage and violation of independence assumptions, training and test sets were created at the patient level rather than the ulcer level. Specifically, a group‐based data splitting strategy was applied, in which all ulcers from the same patient were assigned exclusively to either the training set or the test set. This approach ensured that no patient contributed ulcers to both datasets and prevented inflation of predictive performance estimates.

In the second part of the study, to improve the performance of the existing classification systems to predict unhealing outcomes in outpatient services, we proposed modifications to the WIFI, SINBAD and DiaFORA as follows:

1. *First Proposed WIFI Model*: The original WIFI model was modified by replacing the ischemia and wound components with revised definitions. The ischemia component was refined as peripheral pulses and ABI instead of only ABI index, and the wound depth was redefined using three categories, as score 1 (superficial wound), score 2 (muscle involvement), score 3 (deep wound to tendon, bone or joint).
2. *Second Proposed WIFI Model*: This model only incorporated the revised wound depth categories (as above) into the wound component.

### 2.4 Proposed Modification to the SINBAD Classification Tool

The original SINBAD model was enhanced by including detailed wound location (1: Forefoot—toes, 2: Forefoot—distal, 3: Metatarsal, 4: Hindfoot), replacing the original ischemia component with the Ankle‐Brachial Index (ABI) and the infection component with the IDSA (Infectious Diseases Society of America) classification.

### 2.5 Proposed Modifications to the DiaFORA Classification Tool

1. *First Proposed DiaFORA Model*: The original model was revised by incorporating peripheral vascular disease defined via ABI and infection assessed using the IDSA classification.
2. *Second Proposed DiaFORA Model*: This version added wound dimensions categorised as 1: < 1 cm^2^, 2: Between 1 and 3 cm^2^, 3: > 3 cm^2^.
3. *Third Proposed DiaFORA Model*: This comprehensive model combined all enhancements: addition of peripheral vascular disease (via ABI), infection (via IDSA) and wound location (1‐forefoot: fingers 2‐forefoot: distal 3‐ metatarsal 4‐hindfoot).

Performance of models evaluated using accuracy, AUC, precision (positive predictive value), recall (sensitivity), F1‐score and specificity as follows:

$$\text{Recall or sensitivity}=\frac{\text{True positive}}{\text{True positive}+\text{False Negative}}$$

$$\text{Specificity}=\frac{\text{True Negative}}{\text{False Positive}+\text{True Negative}}$$

$$\text{Precision}=\frac{\text{True positive}}{\text{True positive}+\text{False Positive}}$$

$$\text{Accuracy}=\frac{\text{True positive}+\text{True Negative}}{\text{True positive}+\text{True Negative}+\text{False Positive}+\text{False Negative}}$$

$$F1–\text{Score}=\frac{2*\text{True positive}}{2*\text{True positive}+\text{False Positive}+\text{False Negative}}$$

## 3 Results

This study included 616 DFU for 400 participants, with a mean age of 60.8 (SD = 9.5), 61.3 (SD = 9.3) and 63.5 (SD = 9.9) years in the three groups of healed, unhealed and amputated patients, respectively. The majority of participants were men (66.9%), and within 6 months, the percentages of healing, non‐healing and amputation were 64.6%, 24.5% and 10.9%, respectively. In women, these rates were 82.4%, 12.2% and 5.4%, respectively. The characteristics of studied variables are summarised in Table S1.

In the first step of validating each classification system, we evaluated the relationship between each classification system and outcome using univariate logistic regression. The results indicated, in all classification systems, the chance of unhealed wound increased significantly with increasing severity of wounds in terms of their respective stages. According to univariate logistic regression results, increasing wound severity in all classification systems was significantly associated with higher odds of non‐healing. In the Wagner classification, non‐healing risk increased with deeper tissue penetration and the presence of both infection and gangrene in stages 3 and 4. In the University of Texas (UT) classification, grade 3 wounds penetrating tendon, joint, or bone increased the odds of non‐healing within 6 months (OR = 4.3, 95% CI: 2.8–6.7). Wounds with simultaneous infection and ischemia in the UT system also had a higher risk of non‐healing (OR = 4.4, 95% CI: 2.4–7.8). For the SINBAD system, all components except neuropathy were significantly associated with non‐healing. The aggregate SINBAD score also correlated with increased risk: a score of 5 out of 6 corresponded to OR = 7.4 (95% CI: 3.0–18.3), and a score of 6 corresponded to OR = 10.0 (95% CI: 3.9–25.4). In the PEDIS classification, higher non‐healing risk was observed for more severe stages, including larger wound extent (*p* = 0.012), deeper wounds (*p* < 0.001), critical limb ischemia (*p* < 0.001), deep tissue infection including osteomyelitis (*p* < 0.001) and systemic infection (*p* = 0.020) (Table S2).

The variables of neuropathy (*p* = 0.289), mild infection (*p* = 0.106), previous ulcer or amputation (*p* = 0.200) and PAD (*p* = 0.113) did not show significant relationships with healing status (Table S2). The results of our analysis indicated that the univariate models demonstrated limited performance across the various wound classification systems (Table 1).

In the next step of the performance assessment of the classification systems, we used multivariate models using machine learning algorithms. We compared LASSO and random forest feature selection models with the full model for each classification system (as shown in Tables S4–S10). Details of the selected features for each feature selection method applied to the classification systems are provided in Table S3.

In the next step, we identified the best algorithms for each classification system. These selected algorithms formed the basis for our comparative analysis of the classification systems. Tables S4–S10 present the performance of the six wound classification systems in predicting non‐healing outcomes over 6 months. For each classification system, 10 algorithms were evaluated in both full and reduced models. The best‐performing algorithm for each system was identified by comparing models across multiple indicators, including accuracy, sensitivity (recall), specificity, precision, F1 score and AUC. Greater weight was given to sensitivity and F1 score, as these metrics best reflect the ability to correctly identify non‐healing cases while balancing false positives and false negatives.

For example, in Table S4, the logistic regression model with Random Forest–based feature selection outperformed both the LASSO‐based and full models. It achieved a higher F1 score (67.7 vs. 66.7), accuracy (63.6% vs. 59.5%) and specificity (80.2% vs. 75.2%) compared with the LASSO model and also surpassed the full model in accuracy (77.7% vs. 76.0%), sensitivity (72.4% vs. 60.9%) and specificity (80.2% vs. 77.7%) while using fewer variables. Based on these criteria, the Random Forest–reduced logistic regression model was selected as the optimal algorithm for evaluating the Wagner classification system.

This same selection process was applied to the other classification systems, yielding one optimal algorithm for each. Table 2 summarises these models and allows direct comparison across the six systems. As shown, the SINBAD and UTWCS systems demonstrated the strongest performance overall, with higher sensitivity (74.1 for SINBAD and 75.9 for UTWCS) and F1 scores (71.1 and 71.5, respectively) than the other systems, indicating a superior ability to predict non‐healing cases.

**TABLE 2:** Performance of machine learning algorithms for predicting poor prognosis healing outcome during 6 months.

Wound classification system	Selected classifier	Reduced model	Number of predictors	Accuracy	F1 score	Precision	Recall	Specificity	AUC
Wagner	Logistic regression	Random Forest	20	77.7	67.7	63.6	72.4	80.2	81.0
WIFI	SVM‐linear	LASSO	17	79.3	68.9	67.2	70.7	83.5	83.0
UTWCS	SVM‐linear	LASSO	17	80.4	71.5	67.7	75.9	82.6	83.0
SINBAD	Logistic regression	LASSO	19	80.4	71.1	68.3	74.1	83.5	82.0
IDSA‐PEDIS	Logistic regression	Random Forest	20	77.1	66.1	63.5	69.0	81.0	76.0
PEDIS	Logistic regression	LASSO	18	78.2	68.3	64.6	72.4	81.0	83.0
DiaFORA	Logistic regression	Random Forest	21	76.5	63.2	64.3	62.1	83.5	81.0

Abbreviations: DiaFORA, Diabetic Foot Risk Assessment; IDSA, Infectious Diseases Society of America; LASSO, least absolute shrinkage and selection operator; PEDIS, perfusion, extent, depth/tissue loss, infection and sensation; SINBAD, site, ischemia, neuropathy, bacterial infection and depth; UTWCS, University of Texas Wound Classification System; WIfI, wound, ischemia and foot infection.

Upon comparison of the wound classification systems, we proposed modifications to the criteria for each system to improve predictive performance or simplify implementation. The main modifications focused on the WIFI, SINBAD and DiaFORA systems (Table 3). For example, in the WIFI system, the wound component was replaced with a depth grading system (score 1: superficial, score 2: muscle involvement, score 3: deep wound to tendon, bone, or joint). This change provided a more granular representation of wound severity, which allowed the models to better distinguish between patients with poor versus favorable prognoses according to reporting performance metrics (recall and F1‐score of 77.6% and 72.6% for new model compared to 70.7% and 68.9% of original WIFI model).

**TABLE 3:** Performance of suggested modification for wound classification systems in terms of predicting poor prognosis during 6 months—reduced model (LASSO) using machine learning algorithms.

	Selected classifier	Accuracy	F1 score	Precision	Recall	Specificity	AUC
WIFI‐original model	SVM‐linear	79.3%	68.9%	67.2%	70.7%	83.5%	83.0
WIFI‐model 1	SVM‐linear	79.9	70.0	67.7	72.4	83.5	81.0
WIFI‐model 2	Logistic regression	81.0	72.6	68.2	77.6	82.6	83.0
SINBAD‐original model	SVM‐linear	80.4%	71.1%	68.3%	74.1%	83.5%	82.0
SINBAD‐model 1	SVM‐linear	78.2	66.7	66.1	67.2	83.5	81.0
DiaFORA‐original model	XGBoost	76.0%	63.2%	62.7%	63.8%	81.8%	80.0
DiaFORA‐model 1	SVM‐linear	77.7	66.7	64.5	69.0	81.8	82.0
DiaFORA‐model 2	SVM‐linear	77.1	68.2	62.0	75.9	77.7	84.0
DiaFORA‐model 3	Logistic regression	78.2	66.7	66.1	67.2	83.5	83.0

*Note:* Suggested Modification on original wound classification system adjusting for selected predictors by LASSO feature selection (see Table S3 for selected predictors): *Original model*: WIFI (wound), WIFI (ischemia), WIFI (foot infection), WIFI (gangrene). *WIFI‐model 1*: Original model, replacing WIFI (ischemia) and WIFI (wound), with modified ischemia (using pulse and ABI instead of only ABI) and depth grade as score 1 (superficial wound), score 2 (muscle involvement), score 3 (deep wound to tendon, bone or joint). *WIFI‐model 2*: Original model, replacing WIFI (wound) in original model with depth grade as score 1 (superficial wound), score 2 (muscle involvement), score 3 (deep wound to tendon, bone or joint). *SINBAD‐original model*: Site, Ischemia, Neuropathy, Bacterial Infection, Area and Depth. *SINBAD‐model 1*: Original model, replacing components of site, Ischemia and Bacterial Infection with PEDIS2, ABI and IDSA. *DiaFORA‐original model*: including neuropathy, deformity, peripheral arterial disease or PAD, previous ulcer or lower limb amputation, multiple DFUs, infection, gangrene, bone exposed. *DiaFORA‐model 1*: Original model, replacing components of peripheral arterial disease or PAD and infection with ABI and IDSA. *DiaFORA‐model 2*: Original model + PEDIS2 (as 1 for < 1 cm^2^, 2 for 1–3 cm^2^ and 3 for > 3 cm^2^). *DiaFORA‐model 3*: Original model + PEDIS2 (as 1 for < 1 cm^2^, 2 for 1–3 cm^2^ and 3 for > 3 cm^2^), ulcer site (1‐forefoot: fingers, 2‐forefoot: distal, 3‐ metatarsal, 4‐hindfoot) and, replacing components of peripheral arterial disease or PAD and infection with ABI and IDSA.

Abbreviations: DiaFORA, Diabetic Foot Risk Assessment; LASSO, least absolute shrinkage and selection operator; PEDIS, perfusion, extent, depth/tissue loss, infection and sensation; SINBAD, site, ischemia, neuropathy, bacterial infection, area and depth; UTWCS, University of Texas Wound Classification System; WIfI, wound, ischemia and foot infection.

For the SINBAD system, switching from a binary scoring approach to more detailed component scores did not meaningfully improve predictions, suggesting that additional granularity alone was insufficient without changing key risk predictors. Conversely, modifications to the DiaFORA system—particularly in model 2—incorporated more specific measures of ulcer size (PEDIS2) and peripheral arterial disease (ABI, IDSA), which enhanced the model's ability to identify individuals at high risk for poor outcomes.

Overall, the multivariate models demonstrated that the WIFI model 2 modifications, which replaced the original wound component with depth grading, had the greatest impact on prediction. By providing a finer‐scale measurement of wound severity, the model more accurately assigned risk probabilities, improving discrimination between patients with good versus poor prognoses compared to the original WIFI, SINBAD, or UTWCS systems. Table 3 presents performance metrics (accuracy, F1 score, precision, recall, specificity and AUC) for all systems and their modified versions, highlighting the improvements achieved through targeted feature modifications.

## 4 Discussion

Our analysis provides critical insights into the performance of various wound classification systems, particularly in the context of prognostic prediction. The univariate models assessed in our study demonstrated limited efficacy across all classification systems, reinforcing the notion that relying solely on traditional scoring methods may be insufficient for accurate clinical decision‐making. This highlights the necessity of validating wound classification systems in diverse settings with varying demographic and clinical characteristics, as each system may lack the power to effectively identify cases with poor prognosis, especially given the differences in facility levels and specialised resources.

The challenges faced by different countries due to varying conditions can significantly impact the effectiveness capacity of classification systems. Factors such as inadequate training and supervision of staff in correctly classifying wounds and managing them appropriately, as well as the lack of periodic calibration of diagnostic tools, can hinder performance. Furthermore, socio‐economic factors, such as delays in patients' time to first visit, extended waiting times for specialised services in the public sector, high costs associated with private sector care and insufficient or lacking insurance coverage, underscore the urgent need for prognostic tools tailored to specific settings. IWGDF has emphasised the importance of validating these tools before recommending their implementation for effective communication among healthcare staff [6].

However, the IWGDF guidelines emphasise that none of the currently available classification systems should be used to provide individual patient outcome prognosis. In line with these recommendations, the objective of this study was not to generate individual‐level prognostic predictions for clinical decision‐making. Instead, we evaluated the comparative discriminative performance at the population level of commonly used DFU classification systems for identifying patients at higher risk of non‐healing in an outpatient cohort, using statistical and machine learning approaches.

Based on the results of our study, both the SINBAD and UTWCS classification systems outperformed other classification systems, particularly the WIFI system, in predicting poor prognostic outcomes. In a study similar to ours in Scotland [14], two classification tools, UTWCS and SINBAD, were compared, concluding that they both performed similarly in predicting poor prognosis outcomes. Furthermore, a study in Manchester [15] found that the UTWCS tool exhibited better performance than the Wagner classification. In contrast, other studies [13, 16, 17], indicated that the WIFI system outperformed the others in predicting healing time and provided superior information regarding amputation risk. Additionally, in a study [18], three tools—PEDIS, SINBAD and Wagner—were compared, revealing that PEDIS demonstrated superior diagnostic power compared to the other two tools.

Our results suggest that the relatively weaker performance of the WIFI system can be attributed to two main factors. First, the wound component in the WIFI system is primarily designed for extensive or gangrenous wounds, which were less prevalent in the outpatient clinic setting of this study. Second, the system's effectiveness was limited by the unavailability of specialised equipment, such as transcutaneous oxygen pressure and toe blood pressure measurements, which are essential for accurately assessing ischemia. Moreover, reliance on ABI for ischemia categorization introduced limitations, particularly as many diabetic patients had ABI values > 1.3 due to arterial calcification, masking true ischemia [19]. Furthermore, our ABI device designated values < 0.5 as E1, limiting precision in assessing varying degrees of ischemia in patients with values below 0.5.

Our findings align with recent IWGDF recommendations advocating for classification systems that do not necessitate specialised equipment, such as toe pressure and TcPO2 measurements. Following these recommendations, the SINBAD system stands out as a straightforward and efficient system, requiring only a clinical examination. It provides crucial information for triage by healthcare professionals, facilitating effective communication regarding wound characteristics.

We also proposed modifications to existing classification systems, which yielded promising results. Specifically, in the WIFI system, revising the component related to the wound assessment markedly enhanced the system's predictive capabilities for poor outcomes. The modification of the wound component to a simpler depth classification—designating wounds as 1 for Skin, 2 for Muscle and 3 for Tendon, Joint and Bone—allowed for clearer categorization. As a result, sensitivity improved from 70.7% in the original model to 77.6%, surpassing the performance of all existing systems. The F1 scores and precision metrics also ranked highly, indicating that the modified system was well‐suited for routine outpatient care settings, which typically encounter fewer complex wounds, and can improve feasibility for the healthcare providers operating at these primary care levels and not limit its utility in specialised hospitals and tertiary care centers.

In the present study, peripheral neuropathy was not significantly associated with wound healing outcomes. This finding appears to differ from several previous studies, which identified neuropathy as an independent predictor of non‐healing [20, 21, 22, 23]. However, an important explanation lies in the very high prevalence of neuropathy in our cohort (95.3%), resulting in limited variability and reduced statistical power to detect its independent effect. From a pathophysiological perspective, neuropathy is widely recognised as a key factor in the development of diabetic foot ulcers, primarily through loss of protective sensation, foot deformity, altered pressure distribution, and skin breakdown [24, 25, 26, 27]. Its role in impairing wound healing after ulcer formation, however, is less consistent in the literature. Several studies suggest that neuropathy primarily acts upstream by facilitating ulcer occurrence, whereas peripheral arterial disease and infection play more direct roles in delayed healing and tissue loss. Although some human studies and animal models suggest that neuropathy may adversely affect wound healing through vascular, metabolic, and inflammatory mechanisms, clinical evidence remains heterogeneous [28, 29].

### 4.1 Our Study Had Several Limitations

In our study, ABI is used to assess ischemia in the WIFI classification system, which ABI has known limitations in diabetes due to medial arterial calcification, which can lead to falsely elevated values (> 1.3) and misclassification of ischemia as normal. This limitation may partially explain the weaker performance of ischemia‐dependent systems such as WIFI in this study. The WIFI classification requires more detailed ischemia assessment, ideally including toe pressure or transcutaneous oxygen measurements. However, such measurements were not available in our setting. Since most wound centers in our setting lacked these tools, as a result, ischemia severity may have been underestimated, reducing the predictive performance of WIFI. This limitation underscores the need for classification systems that align with the structural constraints of such facilities.

Also, the apparent inconsistency in the prognostic significance of PAD across classification systems likely reflects differences in ischemia assessment rather than true disagreement. Peripheral arterial disease (PAD) was defined differently across the classification systems included in this study, reflecting their original conceptual frameworks. In the DiaFORA classification, perfusion status represents severe ischemia and was determined based on clinical assessment of peripheral pulses (dorsalis pedis and posterior tibial arteries). The presence of ≤ 1 palpable pedal pulse was defined as PAD. In contrast, other classification systems primarily defined PAD based on ankle–brachial index (ABI) measurements, with values < 0.9 or > 1.3, or absence of palpable pulses considered abnormal.

Another limitation is some laboratory predictors had missing values; we addressed this issue using multiple imputation methods, but residual bias cannot be excluded. Third, class imbalance is a common challenge in prognostic studies of diabetic foot ulcers. Although resampling techniques such as oversampling, undersampling and SMOTE are frequently recommended, their effectiveness depends on data structure and sample size. In our study, resampling approaches did not improve test‐set performance and occasionally degraded model generalisation. The lack of performance improvement with SMOTE may be explained by the moderate degree of imbalance, the relatively limited number of non‐healed cases and the high dimensionality and clinical heterogeneity of the predictor space. In such settings, synthetic samples may not adequately capture clinically meaningful patterns and can introduce noise, leading to reduced generalisation performance. Similar observations have been reported in medical prediction studies with structured clinical data.

Finally, although information on medications was collected, data on the specific time frame and duration of use were not available, limiting our ability to fully assess their potential impact on outcomes.

In this study we didn't assess calibration to develop probabilistic risk scores or treatment thresholds to guide clinical decisions, because our primary goal was to compare the predictive accuracy of different wound classification systems focusing on metrics derived from predicted class labels, including F1 score, sensitivity, specificity and AUC. Additionally, calibration in small datasets poses challenges. Techniques such as Platt scaling, isotonic regression, or cross‐validated calibration require further data splitting, which can reduce the effective sample size, worsen overfitting, or yield unstable calibration curves. Class imbalance can further complicate calibration assessment.

In our study, we proposed modifications to the existing classification systems aimed at improving their performance and enhancing the feasibility of these systems in outpatient services. Future research is essential to validate these modified systems in larger, multicenter studies focused on determining their generalizability and effectiveness in improving patient outcomes.

## 5 Conclusion

In our outpatient sample, the SINBAD and UTWCS classification systems demonstrated the strongest performance when incorporated into predictive models, consistently achieving higher sensitivity and F1 scores compared with other systems. Our proposed modification of the wound component in the WIFI system also improved its performance, suggesting potential applicability for outpatient services and less specialised clinical settings.

## Author Contributions

**Farideh Mostafavi, Yadollah Mehrabi and Seyed Saeed Hashemi Nazari:** conceptualisation, data curation, formal analysis, methodology, software. **Farideh Mostafavi, Mohammad Reza Amini, Melina Rezvani and Mehrangiz Toutounchi:** investigation, project administration. **Farideh Mostafavi:** roles/writing – original draft. **Yadollah Mehrabi and Seyed Saeed Hashemi Nazari:** supervision and validation, visualisation, funding acquisition, resources. **Farideh Mostafavi:** wrote the first draft of the manuscript. **Farideh Mostafavi, Mohammad Reza Amini, Melina Rezvani, Mehrangiz Toutounchi and Seyed Saeed Hashemi Nazari:** approved the final version of the manuscript and writing – review and editing.

## Funding

The authors have nothing to report.

## Ethics Statement

The study was approved by the Shahid Beheshti university of medical science (ethical code: IR.SBMU.PHNS.REC.1402.056).

## Conflicts of Interest

The authors declare no conflicts of interest.

## Supporting information


**Table S1:** Characteristics of study variables.
**Table S2:** Relationship between poor prognosis and wound classification systems using univariate logistic regression.
**Table S3:** Selected feature in reduced models by wound classification systems.
**Table S4:** Performance of the Wagner Wound Classification System in predicting unhealing outcome over a 6‐month evaluation period.
**Table S5:** Performance of the WIFI Wound Classification System in predicting unhealing outcome over a 6‐month evaluation period.
**Table S6:** Performance of the UTWCs Wound Classification System in predicting unhealing outcome over a 6‐month evaluation period.
**Table S7:** Performance of the SINBAD Wound Classification System in predicting unhealing outcome over a 6‐month evaluation period.
**Table S8:** Performance of the IDSA Wound Classification System in predicting unhealing outcome over a 6‐month evaluation period.
**Table S9:** Performance of the PEDIS Wound Classification System in predicting unhealing outcome over a 6‐month evaluation period.
**Table S10:** Performance of the DIaFORA Wound Classification System in predicting unhealing outcome over a 6‐month evaluation period.

## Data Availability

The datasets generated during and/or analysed in the current study are available from the corresponding author upon reasonable request.

## References

[1] Z. G. Abbas , “Managing the Diabetic Foot in Resource‐Poor Settings: Challenges and Solutions,” Chronic Wound Care Management and Research 4 (2017): 135–142.

[2] M. R. Amini , M. R. Mohajeri‐Tehrani , N. Mehrdad , M. Sanjari , M. Aalaa , and N. Alijani , “IWGDF Diagnosis and Treatment of Diabetic Foot Wound Infections: Local Practical Guide,” Tehran University Medical Journal 79, no. 2 (2021): 112–123.

[3] B. M. Schmidt , J. S. Wrobel , M. Munson , G. Rothenberg , and C. M. Holmes , “Podiatry Impact on High‐Low Amputation Ratio Characteristics: A 16‐Year Retrospective Study,” Diabetes Research and Clinical Practice 126 (2017): 272–277. 28288437 10.1016/j.diabres.2017.02.008

[4] L. Prompers , N. Schaper , J. Apelqvist , et al., “Prediction of Outcome in Individuals With Diabetic Foot Ulcers: Focus on the Differences Between Individuals With and Without Peripheral Arterial Disease,” EURODIALE Study Diabetologia 51, no. 747 (2008): 755. 10.1007/s00125-008-0940-0 PMC2292424 18297261

[5] F. Game , “Classification of Diabetic Foot Ulcers,” Diabetes/Metabolism Research and Reviews 32 (2016): 186–194. 26455509 10.1002/dmrr.2746

[6] M. Monteiro‐Soares , E. J. Hamilton , D. A. Russell , et al., “Guidelines on the Classification of Foot Ulcers in People With Diabetes (IWGDF 2023 Update),” Diabetes/Metabolism Research and Reviews 40, no. 3 (2024): e3648. 37179483 10.1002/dmrr.3648

[7] M. Monteiro‐Soares , E. J. Hamilton , D. A. Russell , et al., “Classification of Foot Ulcers in People With Diabetes: A Systematic Review,” Diabetes/Metabolism Research and Reviews 40, no. 3 (2024): e3645. 37132179 10.1002/dmrr.3645

[8] A. Camilleri , A. Gatt , and C. Formosa , “Inter‐Rater Reliability of Four Validated Diabetic Foot Ulcer Classification Systems,” Journal of Tissue Viability 29, no. 4 (2020): 284–290. 32921550 10.1016/j.jtv.2020.09.002

[9] P. Ince , Z. G. Abbas , J. K. Lutale , et al., “Use of the SINBAD Classification System and Score in Comparing Outcome of Foot Ulcer Management on Three Continents,” Diabetes Care 31, no. 5 (2008): 964–967. 18299441 10.2337/dc07-2367

[10] P. Xie , Y. Li , B. Deng , et al., “An Explainable Machine Learning Model for Predicting In‐Hospital Amputation Rate of Patients With Diabetic Foot Ulcer,” International Wound Journal 19, no. 4 (2022): 910–918. 34520110 10.1111/iwj.13691 PMC9013600

[11] R. E. Wright , “Logistic Regression,” in Reading and Understanding Multivariate Statistics, ed. L. G. Grimm and P. R. Yarnold (American Psychological Association, 1995), 217–244.

[12] A. Basu , W. G. Manning , and J. Mullahy , “Comparing Alternative Models: Log vs Cox Proportional Hazard?,” Health Economics 13, no. 8 (2004): 749–765. 15322988 10.1002/hec.852

[13] F. Mostafavi , M. R. Amini , Y. Mehrabi , E. E. Nasli , and S. S. H. Nazari , “Machine Learning Insights Into Amputation Risk: Evaluating Wound Classification Systems in Diabetic Foot Ulcers,” International Wound Journal 22, no. 6 (2025): e70515. 40462388 10.1111/iwj.70515 PMC12134084

[14] G. P. Leese , E. Soto‐Pedre , and C. Schofield , “Independent Observational Analysis of Ulcer Outcomes for SINBAD and University of Texas Ulcer Scoring Systems,” Diabetes Care 44, no. 2 (2021): 326–331. 33288650 10.2337/dc20-1817

[15] S. O. Oyibo , E. B. Jude , I. Tarawneh , H. C. Nguyen , L. B. Harkless , and A. J. M. Boulton , “A Comparison of Two Diabetic Foot Ulcer Classification Systems: The Wagner and the University of Texas Wound Classification Systems,” Diabetes Care 24, no. 1 (2001): 84–88. 11194247 10.2337/diacare.24.1.84

[16] P. Williams , Z. Bakewell , B. Akinlade , and D. A. Russell , “WIfi Scoring: A Reliable Tool for Risk Stratification in the Diabetic Foot Clinic,” Journal of Vascular Societies Great Britain & Ireland 1, no. 3 (2022): 71–76.

[17] P. N. Vera‐Cruz , P. P. Palmes , L. Tonogan , and A. H. Troncillo , “Comparison of WIFi, University of Texas and Wagner Classification Systems as Major Amputation Predictors for Admitted Diabetic Foot Patients: A Prospective Cohort Study,” Malaysian Orthopaedic Journal 14, no. 3 (2020): 114–123. 33403071 10.5704/MOJ.2011.018 PMC7751999

[18] F. Chuan , K. Tang , P. Jiang , B. Zhou , and X. He , “Reliability and Validity of the Perfusion, Extent, Depth, Infection and Sensation (PEDIS) Classification System and Score in Patients With Diabetic Foot Ulcer,” PLoS One 10, no. 4 (2015): e0124739. 25875097 10.1371/journal.pone.0124739 PMC4395335

[19] A. Abouhamda , M. Alturkstani , and Y. Jan , “Lower Sensitivity of Ankle‐Brachial Index Measurements Among People Suffering With Diabetes‐Associated Vascular Disorders: A Systematic Review,” SAGE Open Med 7 (2019): 2050312119835038. 30854203 10.1177/2050312119835038 PMC6399753

[20] G. Leese , C. Schofield , B. McMurray , et al., “Scottish Foot Ulcer Risk Score Predicts Foot Ulcer Healing in a Regional Specialist Foot Clinic,” Diabetes Care 30, no. 8 (2007): 2064–2069. 17519428 10.2337/dc07-0553

[21] B. C. Callaghan , E. Feldman , J. Liu , et al., “Triglycerides and Amputation Risk in Patients With Diabetes: Ten‐Year Follow‐Up in the DISTANCE Study,” Diabetes Care 34, no. 3 (2011): 635–640. 21285390 10.2337/dc10-0878 PMC3041196

[22] T. G. D. Pemayun , R. M. Naibaho , A. N. Radityo , and A. Prasetyo , “Risk Factors for Lower Extremity Amputation in Patients With Diabetic Foot Ulcers: A Hospital‐Based Case‐Control Study,” Diabet Foot Ankle 6 (2015): 29629. 26651032 10.3402/dfa.v6.29629 PMC4673055

[23] E. Ugwu , O. Adeleye , I. Gezawa , I. Okpe , M. Enamino , and I. Ezeani , “Predictors of Lower Extremity Amputation in Patients With Diabetic Foot Ulcer: Findings From MEDFUN, a Multi‐Center Observational Study,” Journal of Foot and Ankle Research 12 (2019): 34. 31223342 10.1186/s13047-019-0345-y PMC6570910

[24] Q. S. Sadriwala , B. S. Gedam , and M. A. Akhtar , “Risk Factors of Amputation in Diabetic Foot Infections,” International Surgery Journal 5, no. 4 (2018): 1399–1402.

[25] D. G. Armstrong , L. A. Lavery , S. Wu , and A. J. M. Boulton , “Evaluation of Removable and Irremovable Cast Walkers in the Healing of Diabetic Foot Wounds: A Randomized Controlled Trial,” Diabetes Care 28, no. 3 (2005): 551–554. 15735186 10.2337/diacare.28.3.551

[26] M. M. J. Diamond , J. E. Sinacore , D. R. Delitto , A. Blair , V. P. Drury , and D. A. Rose , “Total Contact Casting in Treatment of Diabetic Plantar Ulcers. Controlled Clinical Trial,” Diabetes Care 12 (1989): 348–388. 10.2337/diacare.12.6.384 2659299

[27] M. H. Nabuurs‐Franssen , R. Sleegers , M. S. P. Huijberts , et al., “Total Contact Casting of the Diabetic Foot in Daily Practice: A Prospective Follow‐Up Study,” Diabetes Care 28, no. 2 (2005): 243–247. 15677773 10.2337/diacare.28.2.243

[28] A. M. Richards , D. C. Floyd , and G. Terenghi , “Cellular Changes in Denervated Tissue During Wound Healing in a Rat Model,” British Journal of Dermatology 140, no. 6 (1999): 1093–1099. 10354076 10.1046/j.1365-2133.1999.02908.x

[29] N. S. Gibran , Y. C. Jang , F. F. Isik , et al., “Diminished Neuropeptide Levels Contribute to the Impaired Cutaneous Healing Response Associated With Diabetes Mellitus,” Journal of Surgical Research 108, no. 1 (2002): 122–128. 12443724 10.1006/jsre.2002.6525

