# Supplementary material for: Predicting Diabetic Foot Ulcer Outcomes: Machine Learning‐Based Refinement of IWGDF‐Approved Classifications for Outpatient Services
Source: Endocrinol Diabetes Metab. 2026 May 3;9(3):e70193. doi: 10.1002/edm2.70193 (PMC13136067; doi:10.1002/edm2.70193)
Supplement: Supplementary file 1 — Table S1: Characteristics of study variables. Table S2: Relationship between poor prognosis and wound classification systems using univariate logistic regression. Table S3: Selected feature in reduced models by wound classification systems. Table S4: Performance of the Wagner Wound Classification System in predicting unhealing outcome over a 6‐month evaluation period. Table S5: Performance of the WIFI Wound Classification System in predicting unhealing outcome over a 6‐month evaluation period. Table S6: Performance of the UTWCs Wound Classification System in predicting unhealing outcome over a 6‐month evaluation period. Table S7: Performance of the SINBAD Wound Classification System in predicting unhealing outcome over a 6‐month evaluation period. Table S8: Performance of the IDSA Wound Classification System in predicting unhealing outcome over a 6‐month evaluation period. Table S9: Performance of the PEDIS Wound Classification System in predicting unhealing outcome over a 6‐month evaluation period. Table S10: Performance of the DIaFORA Wound Classification System in predicting unhealing outcome over a 6‐month evaluation period. [file EDM2-9-e70193-s001.docx]

**Methods:**

**The neural network architecture:**

The neural network has 3 layers, the input layers, including predictors. In architecture of neural networks two hidden layers were used. The hidden layer performs all kinds of calculations on the features through the input layer and transfers the result to the output layer, which includes healing and amputation outcomes.

We used a leaky rectified linear unit or leaky ReLU for all layers as the activation function. The weights and biases of the neurons are updated based on the output error. This process is known as Back Propagation (BP) which has two forward pass and backward pass approaches. forward pass refers to the forward process from the input layer to the output layer. First, a training item (i.e., an observation) is randomly or sequentially selected from the training set. After passing through the hidden layer, the predicted output is compared with the target variable in the dataset (e.g., 1 = unhealing, 0 = healing). The difference between the predicted and target variable or prediction error is quantified by the loss function. If there is a difference between the predicted and target variable (a person with unhealed ulcer, consider healing), it propagates an error signal. The loss function we used is BCEWithLogitsLoss and because of classes imbalanced of outcome we used positive weight. With the release of the error signal, backward pass is activated so that the calculations are done from the output layer to the input layer and the process of weights updating is done using the gradient and optimizer. Optimizers determine which combination of weights and biases gives it the best chance of producing correct predictions. In this study, the SGD optimizer or Stochastic gradient descent was used with 0.007 as the learning rate. To prevent overfitting, we used regularization techniques such as dropout with a rate of 20%. The number of epochs we used was 300 and the number of batches for training and validation set was 10.

TableS1: characteristics of study variables

| Non-healed  N (%) | variables |  | Non-healed  N (%) | variables |  |
| --- | --- | --- | --- | --- | --- |
| 1(6.3) | Yes | Alcohol consumption | 146(35.4) | Male | Sex |
| 181(30.2) | No |  | 36(17.6) | Female |  |
| 31(35.2) | Current smoker | Smoking | 18(24.3) | illiterate | Education |
| 36(50) | Quit Smoking |  | 59(30.9) | Elementary |  |
| 115(25.2) | Never smoker |  | 15(55.6) | Secondary |  |
| 23(34.8) | Yes | Renal | 55(24.7) | High-school |  |
| 159(28.9) | No |  | 35(34.7) | Academic |  |
| 13(26.5) | Yes | Heart failure | 167(30.4) | Married | Martial statue |
| 169(29.8) | No |  | 15(23.1) | Single |  |
| 42(35.6) | Yes | Bypass | 6(54.5) | No-insurance | Basic Insurance |
| 140(28.1) | No |  | 122(31) | social security |  |
| 64(31.7) | Yes | Stenting or ballooning | 32(23.4) | Health insurance |  |
| 118(28.5) | No |  | 6(20.7) | Armed forces |  |
| 127(30.5) | Yes | Hypertension | 16(35.6) | Others |  |
| 55(27.6) | No |  | 90(30.7) | No | Supplementary insurance |
| 112(27.1) | Yes | Hypercholesteremia | 92(28.5) | Yes |  |
| 70(34.5) | No |  | 143(31.4) | Tehran | Residential area |
| 28(32.2) | Yes | Heart attack | 10(21.3) | Cities of Tehran province |  |
| 154(29.1) | No |  | 8(21.6) | Alborz province |  |
| 26(41.3) | Yes | Stroke | 21(27.3) | Others |  |
| 156(28.2) | No |  | 51(33.3) | Self-employed | Job |
| 144(29.3) | Yes | Retinopathy | 16(41) | Governmental |  |
| 38(30.4) | No |  | 84(36.5) | Retired |  |
| 27(29) | Yes | Thyroid disorders | 26(15.3) | housewife |  |
| 155(29.6) | No |  | 5(20.8) | Unemployed |  |
| 29(35.4) | Yes | Mental disorders | 60(30.8) | No care | Sequence of diabetes visit |
| 153(28.7) | No |  | 58(24.2) | Every three months |  |
| 31(32) | Yes | Intermittent lameness | 29(36.3) | Every six months |  |
| 151(29.1) | No |  | 4(19) | Yearly |  |
| 61.3(0.7) | Mean (SD) | Age | 31(38.8) | More than a year |  |
| 17.3(0.6) | Mean (SD) | Diabetes duration(year) | 60(30.9) | No care | Diabetes center |
| 177.3(5.7) | Mean (SD) | FBS | 39(23.5) | Private sector |  |
| 8.3(0.1) | Mean (SD) | Hemoglobin A1C | 83(32.4) | Government sector |  |
| 165(6.3) | Mean (SD) | TG | 64(27.1) | Oral medicine | Diabetes medication |
| 148(2.7) | Mean (SD) | Col | 51(38.6) | Insulin |  |
| 80.7(1.1) | Mean (SD) | LDL | 60(25.9) | Both |  |
| 40.2(0.6) | Mean (SD) | HDL | 7(43.8) | None |  |
| 13.1(0.1) | Mean (SD) | Hemoglobin | 119(26.9) | Yes | Statins intake |
| 1.3(0.0) | Mean (SD) | Creatinine | 63(36.4) | No |  |
| 59.9(1.7) | Mean (SD) | eGFR | 127(31.4) | Yes | Anti platelet intake |
| 36.1(1.8) | Mean (SD) | Time to first visit(day) | 55(26.1) | No |  |
| 27.2(0.3) | Mean (SD) | BMI |  |  |  |

Table S2: Relationship between poor prognosis and wound classification systems using univariate logistic regression

|  |  | Total number in a category | Number of poor prognosis (%) | Odds ratio (95%conf. interval) | P-value |
| --- | --- | --- | --- | --- | --- |
| Wagner |  |  |  |  |  |
|  | 1 | 382 | 77(20.2) | Ref |  |
|  | 2 | 115 | 43(37.4) | 2.4(1.5,3.8) | <0.001 |
|  | 3 | 107 | 50(46.7) | 3.5(2.2,5.5) | <0.001 |
|  | 4 | 12 | 12(100) | 47.5(6.1,371.2) | <0.001 |
| UTWCS-grade |  |  |  |  |  |
|  | 1 | 382 | 77(20.2) | Ref |  |
|  | 2 | 115 | 43(37.4) | 2.4(1.5,3.7) | <0.001 |
|  | 3 | 119 | 62(52.1) | 4.3(2.8,6.7) | <0.001 |
| UTWCS-stage |  |  |  |  |  |
|  | 1 | 217 | 44(20.3) | Ref |  |
|  | 2 | 295 | 91(30.8) | 1.7(1.2,2.6) | 0.008 |
|  | 3 | 32 | 9(28.1) | 1.5(0.6,3.5) | 0.314 |
|  | 4 | 72 | 38(52.8) | 4.4(2.4,7.8) | <0.001 |
| IDSA |  |  |  |  |  |
|  | 0 | 247 | 55(22.3) | Ref |  |
|  | 1 | 113 | 17(15) | 0.6(0.3,1.1) | 0.114 |
|  | 2 | 199 | 88(44.2) | 2.8(1.8,4.2) | <0.001 |
|  | 3 | 57 | 22(38.6) | 2.2(1.2,4.0) | 0.012 |
| SINBAD1- Site |  |  |  |  |  |
|  | 0 | 517 | 140(27.1) | Ref |  |
|  | 1 | 99 | 42(42.4) | 2.0(1.27,3.1) | 0.002 |
| SINBAD2- Ischemia | |  | 0 |  |  |
|  | 0 | 513 | 135(26.3) | Ref |  |
|  | 1 | 103 | 47(45.6) | 2.3(1.5,3.6) | <0.001 |
| SINBAD3- Neuropathy | |  | 0 |  |  |
|  | 0 | 29 | 6(20.7) | Ref |  |
|  | 1 | 587 | 176(30) | 1.6(0.6,4.1) | 0.289 |
| SINBAD4- Bacterial infection | |  | #VALUE! |  |  |
|  | 0 | 214 | 48(22.4) | Ref |  |
|  | 1 | 402 | 134(33.3) | 1.7(1.2,2.5) | 0.005 |
| SINBAD5- Area |  |  |  |  |  |
|  | 0 | 246 | 58(23.6) | Ref |  |
|  | 1 | 370 | 124(33.5) | 1.6(1.1,2.3) | 0.008 |
| SINBAD6- Depth |  |  |  |  |  |
|  | 0 | 383 | 77(20.1) | Ref |  |
|  | 1 | 233 | 105(45.1) | 3.2(2.3,4.7) | <0.001 |
| SINBAD (sum) |  |  |  |  |  |
|  | 1 | 6 | 14(14.7) | Ref |  |
|  | 2 | 89 | 34(23.1)) | 1.7(0.9,3.4) | 0.113 |
|  | 3 | 147 | 46(26.0) | 2.0(1.0,39) | 0.035 |
|  | 4 | 177 | 51(37.8) | 3.5(1.8,6.8) | <0.001 |
|  | 5 | 135 | 18(56.2) | 7.4(3.0,18.3) | <0.001 |
|  | 6 | 32 | 19(63.3) | 10.0(3.9,25.4) | <0.001 |
| PEDIS1- Perfusion |  |  |  |  |  |
|  | 0 | 416 | 102(24.5) | Ref |  |
|  | 1 | 88 | 23(26.1) | 1.1(0.6,1.8) | 0.750 |
|  | 2 | 112 | 57(50.9) | 3.2(2.0,4.9) | <0.001 |
| PEDIS2- Extent |  |  |  |  |  |
|  | 1 | 234 | 53(22.6) | Ref |  |
|  | 2 | 145 | 50(34.5) | 1.8(1.1,2.8) | 0.012 |
|  | 3 | 237 | 79(33.3) | 1.7(1.1,2.6) |  |
| PEDIS3-Depth |  |  |  |  |  |
|  | 1 | 382 | 77(20.2) | Ref |  |
|  | 2 | 116 | 44(37.9) | 2.4(1.5,3.8) | <0.001 |
|  | 3 | 118 | 61(51.7) | 4.2(2.7,6.6) | <0.001 |
| PEDIS4-Infection |  |  |  |  |  |
|  | 0 | 247 | 55(22.3) | Ref |  |
|  | 1 | 113 | 17(15) | 0.6(0.3,1.1) | 0.106 |
|  | 2 | 199 | 88(44.2) | 2.8(1.9,4.2) | <0.001 |
|  | 3 | 57 | 22(38.6) | 2.1(1.1,4.0) | 0.020 |
| PEDIS5- Sensation |  |  |  |  |  |
|  | 0 | 29 | 6(20.7) | Ref |  |
|  | 1 | 587 | 176(30) | 1.6(0.6,4.1) | 0.289 |
| DiaFORA1- Neuropathy | |  |  |  |  |
|  | 0 | 29 | 6(20.7) | Ref |  |
|  | 1 | 587 | 176(30) | 1.6(0.6,4.1) | 0.289 |
| DiaFORA2- Deformity | |  |  |  |  |
|  | 0 | 418 | 104(24.9) | Ref |  |
|  | 1 | 198 | 78(39.4) | 2.0(1.4,2.8) | <0.001 |
| DiaFORA3- peripheral arterial disease | |  |  |  |  |
|  | 0 | 236 | 58(24.6) | Ref |  |
|  | 1 | 380 | 124(32.6) | 1.5(1.0,2.1) | 0.034 |
| DiaFORA4- Previous DFU/Amputation | |  |  |  |  |
|  | 0 | 192 | 50(26) | Ref |  |
|  | 1 | 424 | 132(31.1) | 1.3(0.8,1.9) | 0.200 |
| DiaFORA6-Infection | |  |  |  |  |
|  | 0 | 410 | 107(26.1) | Ref |  |
|  | 1 | 206 | 75(36.4) | 1.6(1.1,2.3) | 0.008 |
| DiaFORA7- necrosis or gangrene | |  |  |  |  |
|  | 0 | 500 | 138(27.6) | Ref |  |
|  | 1 | 116 | 44(37.9) | 1.6(1.0,2.4) | 0.035 |
| DiaFORA8- probe to bone | |  |  |  |  |
|  | 0 | 500 | 122(24.4) | Ref |  |
|  | 1 | 116 | 60(51.7) | 3.3(2.2,5.0) | <0.001 |
| WIFI1-Infection |  |  |  |  |  |
|  | 0 | 248 | 55(22.2) |  |  |
|  | 1 | 114 | 17(14.9) | 0.6(0.3,1.1) | 0.114 |
|  | 2 | 101 | 90(89.1) | 2.8(1.8,4.2) | <0.001 |
|  | 3 | 53 | 20(37.7) | 2.2(1.2,4.0) | 0.012 |
| WIFI2- Ischemia |  |  |  |  |  |
|  | 0 | 540 | 140(25.9) | Ref |  |
|  | 1 | 5 | 3(60) | 4.3(0.7,25.9) | 0.113 |
|  | 3 | 71 | 39(54.9) | 3.5(2.1,5.7) | <0.001 |
| WIFI3-wound |  |  |  |  |  |
|  | 1 | 468 | 105(22.4) | Ref |  |
|  | 2 | 136 | 70(51.5) | 3.7(2.4,5.5) | <0.001 |
|  | 3 | 12 | 7(58.3) | 4.8(1.5,15.5) | 0.008 |
| WIFI3-gangrene |  |  |  |  |  |
|  | 0 | 249 | 53(21.3) | Ref |  |
|  | 1 | 355 | 117(33) | 1.8(1.2,2.6) | 0.002 |
|  | 2 | 10 | 10(100) | 36.9(4.6,295.4) | 0.001 |
|  | 3 | 2 | 2(100) | Empty healed case |  |
| UTWCS: University of Texas Wound Classification System, SINBAD: Site, Ischemia, Neuropathy, Bacterial Infection and Depth, IDSA: Infectious Diseases Society of America, PEDIS: perfusion, extent, depth/tissue loss, infection, and sensation, DiaFORA: Diabetic Foot Risk Assessment | | | | | |

Table S3: selected feature in reduced models by wound classification systems

|  | Wagner | | WIFI | | UTWCS | | SINBAD | | IDSA | | PEDIS | | DiaFORA | |
| --- | --- | --- | --- | --- | --- | --- | --- | --- | --- | --- | --- | --- | --- | --- |
| Model | LASOO | RF | LASOO | RF | LASOO | RF | LASOO | RF | LASOO | RF | LASOO | RF | LASOO | RF |
| Number of variables | 17 | 20 | 17 | 16 | 17 | 15 | 19 | 20 | 16 | 20 | 18 | 20 | 23 | 20 |
| Time to first specialist visit | × | × | × | × | × | × | × | × | × | × | × | × | × | × |
| Body mass index | × | × | × | × | × | × | × | × | × | × | × | × | × | × |
| Hemoglobin | × | × | × | × | × | × |  | × |  | × |  | × |  | × |
| Hypercholesteromia |  | × |  | × |  | × |  | × |  | × |  | × |  | × |
| eGFR |  | × |  | × |  | × |  | × |  | × |  | × |  | × |
| Triglycerides |  | × | × | × |  | × |  | × |  | × | × | × | × | × |
| LDL |  | × |  | × |  | × |  | × |  | × |  | × | × | × |
| HDL | × | × |  | × |  | × |  | × | × | × |  | × |  | × |
| Hemoglobin A1c | × | × | × | × |  | × | × | × |  | × |  | × |  | × |
| Fasting blood sugar | × | × | × | × | × | × | × | × | × | × | × | × | × | × |
| Blood creatinine |  | × |  | × |  | × |  | × |  | × |  | × |  | × |
| Age |  | × |  | × |  | × |  | × |  | × |  | × |  | × |
| Sex |  | × |  |  |  |  |  | × |  | × |  | × |  |  |
| Occupation-housewife | × | × | × |  | × |  | × |  | × |  | × | × | × | × |
| Duration of diabetes | × | × | × | × | × | × | × | × | × | × | × | × | × | × |
| Public sector diabetes care | × | × | × |  | × |  | × |  | × | × | × |  | × |  |
| No diabetes care |  |  | × |  | × |  | × |  | × |  | × |  | × |  |
| Diabetes care every three months | × |  |  |  | × |  |  |  |  |  |  |  |  |  |
| Diabetes care every six months |  |  |  |  |  |  | × |  |  |  | × |  | × |  |
| Diabetes care for more than one year | × |  | × |  | × |  | × |  | × | × | × |  | × |  |
| Education |  | × |  |  |  |  |  |  |  | × |  | × |  |  |
| Basic insurance type-Medical services |  |  |  |  | × |  |  |  |  |  |  |  |  |  |
| Basic insurance type-Social security | × |  | × |  |  |  | × |  | × |  | × |  | × |  |
| Residence in Tehran | × |  | × |  | × |  | × |  | × |  | × |  | × |  |
| Drug use |  |  |  |  |  |  |  |  |  |  |  |  |  |  |
| Quit smoking | × |  |  |  | × |  | × |  | × |  |  |  | × |  |
| Years of smoking | × |  | × |  | × |  | × |  | × |  | × |  | × |  |
| oral medication and insulin |  |  |  |  |  |  |  |  |  |  |  |  | × |  |
| Statins intake |  |  |  |  |  |  |  |  |  |  |  |  | × |  |
| Psychiatric disorders | × | × |  |  | × |  | × |  | × | × | × |  | × |  |
| Heart attack |  | × |  |  |  |  |  |  |  | × |  |  |  |  |
| High blood pressure |  |  |  |  |  |  |  |  | × |  |  |  |  |  |
| Wagner | × | × |  |  |  |  |  |  |  |  |  |  |  |  |
| University of Texasstage |  |  |  |  | × | × |  |  |  |  |  |  |  |  |
| University of Texasgrade |  |  |  |  | × | × |  |  |  |  |  |  |  |  |
| WIFI (ischemia) |  |  | × | × |  |  |  |  |  |  |  |  |  |  |
| WIFI (infection) |  |  | × | × |  |  |  |  |  |  |  |  |  |  |
| WIFI (wound) |  |  | × | × |  |  |  |  |  |  |  |  |  |  |
| SINBAD1 (site) |  |  |  |  |  |  |  | × |  |  |  |  |  |  |
| SINBAD2 (ischemia) |  |  |  |  |  |  | × | × |  |  |  |  |  |  |
| SINBAD3 (neuropathy) |  |  |  |  |  |  |  | × |  |  |  |  |  |  |
| SINBAD4 (bacterial infection) |  |  |  |  |  |  | × | × |  |  |  |  |  |  |
| SINBAD5 (area) |  |  |  |  |  |  | × | × |  |  |  |  |  |  |
| SINBAD6 (depth) |  |  |  |  |  |  | × | × |  |  |  |  |  |  |
| DiaFORA1 (neuropathy) |  |  |  |  |  |  |  |  |  |  |  |  |  |  |
| DiaFORA2 (foot deformity) |  |  |  |  |  |  |  |  |  |  |  |  | × | × |
| DiaFORA3 (peripheral vascular disease) |  |  |  |  |  |  |  |  |  |  |  |  | × | × |
| DiaFORA4 (history of previous ulcer or amputation) |  |  |  |  |  |  |  |  |  |  |  |  | × | × |
| DiaFORA6 (infection) |  |  |  |  |  |  |  |  |  |  |  |  |  | × |
| DiaFORA7 (gangrene or necrosis) |  |  |  |  |  |  |  |  |  |  |  |  | × | × |
| DiaFORA8 (wound penetration into bone) |  |  |  |  |  |  |  |  |  |  |  |  | × | × |
| PEDIS1 (perfusion) |  |  |  |  |  |  |  |  |  |  | × | × |  |  |
| Wound extent (PEDIS2) |  |  |  |  |  |  |  |  |  |  | × | × |  |  |
| PEDIS3 (wound depth) |  |  |  |  |  |  |  |  |  |  | × | × |  |  |
| PEDIS4 (infection) |  |  |  |  |  |  |  |  |  |  | × | × |  |  |
| PEDIS5 (sensation) |  |  |  |  |  |  |  |  |  |  |  |  |  |  |
| IDSA |  |  |  |  |  |  |  |  | × | × |  |  |  |  |

**Performance of the Wagner Wound Classification System in Predicting Non-Healing Outcomes**

Table S4 presents the performance of the Wagner Wound Classification System in predicting non-healing outcomes over a six-month follow-up period. Among the tested models, the Radial Basis Function (RBF) Support Vector Machine (SVM) showed the best performance in both the full model and the reduced LASSO model, while logistic regression performed best in the reduced Random Forest model.

When comparing these models based on their best-performing algorithms, the logistic regression model with Random Forest-based feature selection outperformed both the LASSO-based and full models. Specifically, it achieved a higher F1 score (67.7 vs. 66.7), accuracy (63.6% vs. 59.5%), and specificity (80.2% vs. 75.2%) compared to the LASSO model. It also surpassed the full model in accuracy (77.7% vs. 76.0%), sensitivity (72.4% vs. 60.9%), and specificity (80.2% vs. 77.7%), while using fewer variables.

Based on these findings, the logistic regression model with the reduced Random Forest feature set was selected as the optimal model for evaluating and comparing the performance of the Wagner Wound Classification System against other classification systems, as summarized in Table S4.

Table S4: Performance of the Wagner Wound Classification System in Predicting unhealing Outcome Over a Six-Month Evaluation Period

|  | Machine learning algorithm | number of Variable | Accuracy | F1 score | precision | Sensitivity | specificity | AUC |
| --- | --- | --- | --- | --- | --- | --- | --- | --- |
|  | Full model | | |  |  |  |  |  |
| 1 | Random Forest | 64 | 72.1 | 34.5 | 44.4 | 62.5 | 90.1 | 76.0 |
| 2 | Decision tree | 64 | 64.8 | 41.4 | 43.2 | 45.3 | 76.0 | 59.0 |
| 3 | XGBoost | 64 | **76.5** | 58.6 | 61.8 | 65.4 | 85.1 | 80.0 |
| 4 | Support Vector Machine - RBF | 64 | 76.0 | 72.4 | **66.1** | 60.9 | 77.7 | **83.0** |
| 5 | Linear support vector machine | 64 | 70.9 | 63.8 | 58.7 | 54.4 | 74.4 | 77.0 |
| 6 | Gaussian Process | 64 | 70.4 | 36.2 | 44.2 | 56.8 | 86.8 | 68.0 |
| 7 | Simple Bayes | 64 | 39.7 | **86.2** | 48.1 | 33.3 | 17.4 | 72.0 |
| 8 | K Nearest neighbor | 64 | 74.3 | 31.0 | 43.9 | **75.0** | **95.0** | 67.0 |
| 9 | Logistic regression | 64 | 77.7 | 65.5 | 65.5 | 65.5 | 83.5 | 80.0 |
| 10 | Neural networks | 64 | 72.1 | 56.1 | 57.1 | 55.2 | 80.0 | 67.7 |
|  | Reduced LASSO model | | | |  |  |  |  |
| 1 | Random Forest | 17 | 76.0 | 56.6 | 68.3 | 48.3 | 89.3 | 77.0 |
| 2 | Decision tree | 17 | 59.8 | 47.8 | 41.3 | 56.9 | 61.2 | 60.0 |
| 3 | XGBoost | 17 | **78.8** | **68.3** | 66.1 | 70.7 | 82.6 | 80.0 |
| 4 | Support Vector Machine - RBF | 17 | 75.4 | 66.7 | 59.5 | **75.9** | 75.2 | 80.0 |
| 5 | Linear support vector machine | 17 | 76.0 | 66.1 | 60.9 | 72.4 | 77.7 | 80.0 |
| 6 | Gaussian Process | 17 | 74.3 | 45.2 | **73.1** | 32.8 | **94.2** | 77.0 |
| 7 | Simple Bayes | 17 | 72.1 | 54.5 | 57.7 | 51.7 | 81.8 | 77.0 |
| 8 | K Nearest neighbor | 17 | 70.9 | 39.5 | 60.7 | 29.3 | 90.9 | 71.0 |
| 9 | Logistic regression | 17 | 76.0 | 66.1 | 60.9 | 72.4 | 77.7 | **81.0** |
| 10 | Neural networks | 17 | 73.2 | 51.0 | 62.5 | 43.1 | 88.0 | 65.3 |
|  | Reduced Random Forest Model | | | |  |  |  |  |
| 1 | Random Forest | 20 | 70.9 | 40.9 | 60.0 | 31.0 | 90.1 | 74.0 |
| 2 | Decision tree | 20 | 62.6 | 44.6 | 42.9 | 46.6 | 70.2 | 58.0 |
| 3 | XGBoost | 20 | 68.7 | 52.5 | 51.7 | 53.4 | 76.0 | 77.0 |
| 4 | Support Vector Machine - RBF | 20 | 67.6 | 61.8 | 50.0 | 81.0 | 61.2 | 81.0 |
| 5 | Linear support vector machine | 20 | 76.0 | 65.0 | 61.5 | 69.0 | 79.3 | 80.0 |
| 6 | Gaussian Process | 20 | 72.6 | 38.0 | 71.4 | 25.9 | 95.0 | 71.0 |
| 7 | Simple Bayes | 20 | 79.3 | 63.4 | 74.4 | 55.2 | 90.9 | 81.0 |
| 8 | K Nearest neighbor | 20 | 71.5 | 44.0 | 60.6 | 34.5 | 89.3 | 68.0 |
| 9 | Logistic regression | 20 | **77.7** | **67.7** | **63.6** | **72.4** | **80.2** | **81.0** |
| 10 | Neural networks | 20 | 69.3 | 42.1 | 54.0 | 34.5 | 60.2 | 60.0 |
| LASSO: Least Absolute Shrinkage and Selection Operator, RBF : Radial Basis Function | | | | | | | | |

**Performance of the WIFI Wound Classification System in Predicting Non-Healing Outcomes**

Table S5 presents the performance of the WIFI Wound Classification System in predicting non-healing outcomes over a six-month follow-up period. Among the algorithms evaluated, XGBoost achieved the best performance in the full model, while the linear Support Vector Machine (SVM) and logistic regression performed best in the reduced LASSO and reduced Random Forest models, respectively.

When comparing the models based on their top-performing algorithms, the linear SVM in the LASSO model outperformed the logistic regression in the Random Forest model in terms of accuracy (79.3% vs. 78.8%), precision (67.2% vs. 65.6%), specificity (83.5% vs. 81.8%), and area under the curve (AUC) (83.0 vs. 80.0), with comparable F1 scores. Although the full model exhibited slightly better overall performance, the LASSO-based model used fewer variables.

Therefore, due to its strong performance and model simplicity, the linear SVM in the reduced LASSO model was selected for comparing the WIFI Wound Classification System with other classification systems.

Table S5: Performance of the WIFI Wound Classification System in Predicting unhealing Outcome Over a Six-Month Evaluation Period

|  | Machine learning algorithm | number of Variable | Accuracy | F1 score | precision | Sensitivity | specificity | AUC |
| --- | --- | --- | --- | --- | --- | --- | --- | --- |
|  | Full model |  |  |  |  |  |  |  |
| 1 | Random Forest | 67 | 72.6 | 31.0 | **84.6** | 19.0 | **98.3** | 79.0 |
| 2 | Decision tree | 67 | 58.7 | 35.1 | 35.7 | 34.5 | 70.2 | 53.0 |
| 3 | XGBoost | 67 | **81.6** | **71.8** | 71.2 | 72.4 | 86.0 | 82.0 |
| 4 | Support Vector Machine - RBF | 67 | 74.3 | 54.9 | 63.6 | 48.3 | 86.8 | 78.0 |
| 5 | Linear support vector machine | 67 | 77.1 | 66.1 | 63.5 | 69.0 | 81.0 | **84.0** |
| 6 | Gaussian Process | 67 | 68.2 | 40.0 | 51.4 | 32.8 | 85.1 | 64.0 |
| 7 | Simple Bayes | 67 | 39.7 | 48.1 | 33.3 | **86.2** | 17.4 | 73.0 |
| 8 | K Nearest neighbor | 67 | 71.5 | 37.0 | 65.2 | 25.9 | 93.4 | 66.0 |
| 9 | Logistic regression | 67 | 76.0 | 61.3 | 64.2 | 58.6 | 84.3 | **84.0** |
| 10 | Neural networks | 67 | 73.7 | 48.3 | 66.7 | 37.9 | 91.0 | 64.4 |
|  | Reduced LASSO model | | | |  |  |  |  |
| 1 | Random Forest | 17 | 74.3 | 47.7 | 70.0 | 36.2 | 92.6 | 79.0 |
| 2 | Decision tree | 17 | 62.6 | 41.7 | 42.1 | 41.4 | 72.7 | 56.0 |
| 3 | XGBoost | 17 | 78.8 | 67.2 | 67.2 | 67.2 | 84.3 | 82.0 |
| 4 | Support Vector Machine - RBF | 17 | 73.2 | 54.7 | 60.4 | 50.0 | 84.3 | 77.0 |
| 5 | Linear support vector machine | 17 | **79.3** | **68.9** | 67.2 | 70.7 | 83.5 | **83.0** |
| 6 | Gaussian Process | 17 | 73.7 | 39.0 | 78.9 | 25.9 | 96.7 | 79.0 |
| 7 | Simple Bayes | 17 | 79.3 | 64.8 | 72.3 | 58.6 | 89.3 | 81.0 |
| 8 | K Nearest neighbor | 17 | 73.7 | 49.5 | 65.7 | 39.7 | 90.1 | 72.0 |
| 9 | Logistic regression | 17 | 74.3 | 64.6 | 58.3 | **72.4** | 75.2 | **83.0** |
| 10 | Neural networks | 17 | 77.6 | 55.5 | **78.1** | 43.1 | **94.0** | 68.6 |
|  | Reduced Random Forest Model | | | |  |  |  |  |
| 1 | Random Forest | 16 | 72.6 | 42.4 | 66.7 | 31.0 | 92.6 | 78.0 |
| 2 | Decision tree | 16 | 54.7 | 30.8 | 30.5 | 31.0 | 66.1 | 48.0 |
| 3 | XGBoost | 16 | 74.3 | 60.3 | 60.3 | 60.3 | 81.0 | 78.0 |
| 4 | Support Vector Machine - RBF | 16 | 54.2 | 37.9 | 33.8 | 43.1 | 59.5 | 55.0 |
| 5 | Linear support vector machine | 16 | 53.1 | 51.7 | 38.8 | **77.6** | 41.3 | 76.0 |
| 6 | Gaussian Process | 16 | 65.4 | 32.6 | 44.1 | 25.9 | 84.3 | 62.0 |
| 7 | Simple Bayes | 16 | 77.1 | 63.1 | 66.0 | 60.3 | 85.1 | 79.0 |
| 8 | K Nearest neighbor | 16 | 63.7 | 17.7 | 33.3 | 12.1 | 88.4 | 59.0 |
| 9 | Logistic regression | 16 | **78.8** | **68.9** | 65.6 | 72.4 | 81.8 | **80.0** |
| 10 | Neural networks | 16 | 76.0 | 48.2 | **80.0** | 34.4 | **96.0** | 65.2 |
| LASSO: Least Absolute Shrinkage and Selection Operator, WIFI : Wound Ischemia and foot Infection , RBF: Radial Basis Function | | | | | | | | |

**Performance of the University of Texas Wound Classification System in Predicting Non-Healing Outcomes**

Table S6 presents the performance of the University of Texas Wound Classification System in predicting non-healing outcomes over a six-month follow-up period. The linear Support Vector Machine (SVM) algorithm showed the best performance in both the full model and the reduced LASSO model, while logistic regression performed best in the reduced Random Forest model.

When comparing the models based on their top-performing algorithms, the LASSO model consistently outperformed the Random Forest model across all performance metrics. Furthermore, it achieved higher F1 score (71.5 vs. 70.6) and sensitivity (75.9% vs. 72.4%) compared to the best-performing algorithm in the full model. Given its superior performance with fewer input variables, the linear SVM in the reduced LASSO model was selected for comparing the University of Texas Wound Classification System with other wound classification systems.

Table S6: Performance of the UTWCs Wound Classification System in Predicting unhealing Outcome Over a Six-Month Evaluation Period

|  | Machine learning algorithm | number of Variable | Accuracy | F1 score | precision | Sensitivity | specificity | AUC |
| --- | --- | --- | --- | --- | --- | --- | --- | --- |
|  | Full model | | |  |  |  |  |  |
| 1 | Random Forest | 65 | 72.1 | 28.6 | 83.3 | 17.2 | **98.3** | 79.0 |
| 2 | Decision tree | 65 | 63.1 | 45.0 | 43.5 | 46.6 | 71.1 | 59.0 |
| 3 | XGBoost | 65 | 74.3 | 59.6 | 60.7 | 58.6 | 81.8 | 80.0 |
| 4 | Support Vector Machine - RBF | 65 | 72.6 | 50.5 | 61.0 | 43.1 | 86.8 | 80.0 |
| 5 | Linear support vector machine | 65 | **80.4** | **70.6** | **68.9** | 72.4 | 84.3 | **83.0** |
| 6 | Gaussian Process | 65 | 73.2 | 52.0 | 61.9 | 44.8 | 86.8 | 71.0 |
| 7 | Simple Bayes | 65 | 39.7 | 48.1 | 33.3 | **86.2** | 17.4 | 72.0 |
| 8 | K Nearest neighbor | 65 | 73.2 | 45.5 | 66.7 | 34.5 | 91.7 | 69.0 |
| 9 | Logistic regression | 65 | 77.7 | 64.9 | 66.1 | 63.8 | 84.3 | 81.0 |
| 10 | Neural networks | 65 | 73.2 | 48.9 | 63.9 | 39.6 | 89.0 | 64.4 |
|  | Reduced LASSO model | | | |  |  |  |  |
| 1 | Random Forest | 17 | 72.6 | 44.9 | 64.5 | 34.5 | 90.9 | 75.0 |
| 2 | Decision tree | 17 | 63.1 | 52.2 | 45.0 | 62.1 | 63.6 | 65.0 |
| 3 | XGBoost | 17 | 76.0 | 62.6 | 63.2 | 62.1 | 82.6 | 79.0 |
| 4 | Support Vector Machine - RBF | 17 | 75.4 | 55.1 | 67.5 | 46.6 | 89.3 | 79.0 |
| 5 | Linear support vector machine | 17 | **80.4** | **71.5** | 67.7 | **75.9** | 82.6 | **83.0** |
| 6 | Gaussian Process | 17 | 75.4 | 50.0 | 73.3 | 37.9 | 93.4 | 80.0 |
| 7 | Simple Bayes | 17 | 72.1 | 54.5 | 57.7 | 51.7 | 81.8 | 76.0 |
| 8 | K Nearest neighbor | 17 | 72.1 | 47.9 | 60.5 | 39.7 | 87.6 | 72.0 |
| 9 | Logistic regression | 17 | 77.1 | 67.2 | 62.7 | 72.4 | 79.3 | 82.0 |
| 10 | Neural networks | 17 | 76.0 | 52.7 | **72.7** | 41.4 | **93.0** | 67.0 |
|  | Reduced Random Forest Model | | | | |  |  |  |
| 1 | Random Forest | 15 | 69.3 | 39.6 | 54.5 | 31.0 | 87.6 | 74.0 |
| 2 | Decision tree | 15 | 65.4 | 44.6 | 46.3 | 43.1 | 76.0 | 59.0 |
| 3 | XGBoost | 15 | 67.6 | 52.5 | 50.0 | 55.2 | 73.6 | 76.0 |
| 4 | Support Vector Machine - RBF | 15 | 57.5 | 56.8 | 42.4 | **86.2** | 43.8 | **77.0** |
| 5 | Linear support vector machine | 15 | 74.3 | 64.1 | 58.6 | 70.7 | 76.0 | **77.0** |
| 6 | Gaussian Process | 15 | 72.6 | 39.5 | **69.6** | 27.6 | 94.2 | 75.0 |
| 7 | Simple Bayes | 15 | 75.4 | 54.2 | 68.4 | 44.8 | 90.1 | 76.0 |
| 8 | K Nearest neighbor | 15 | 74.9 | 54.5 | 65.9 | 46.6 | 88.4 | 73.0 |
| 9 | Logistic regression | 15 | **74.9** | **64.6** | 59.4 | 70.7 | 76.9 | 76.0 |
| 10 | Neural networks | 15 | 72.6 | 48.4 | 62.2 | 39.6 | 100 | 50.0 |
| LASSO: Least Absolute Shrinkage and Selection Operator, RBF: Radial Basis Function | | | | | | | | |

**Performance of the SINBAD Wound Classification System in Predicting Non-Healing Outcomes: Multivariate Model**

Table S7 presents the performance of the SINBAD wound classification system in predicting non-healing outcomes over a six-month follow-up period. Logistic regression was identified as the best-performing algorithm across the full model, the reduced LASSO model, and the reduced Random Forest model.

When comparing models based on their respective best-performing algorithms, the reduced LASSO model outperformed the reduced Random Forest model in all performance metrics. Additionally, it achieved a higher F1 score (71.1 vs. 67.9) and greater sensitivity (74.1% vs. 65.5%) compared to the full model. Given its improved performance and reduced number of variables, logistic regression in the reduced LASSO model was selected for comparing the SINBAD wound classification system with other classification systems.

Table S7: Performance of the SINBAD Wound Classification System in Predicting unhealing Outcome Over a Six-Month Evaluation Period

|  | Machine learning algorithm | number of Variable | Accuracy | F1 score | precision | Sensitivity | specificity | AUC |
| --- | --- | --- | --- | --- | --- | --- | --- | --- |
|  | Full model |  |  |  |  |  |  |  |
| 1 | Random Forest | 69 | 72.6 | 32.9 | 80.0 | 20.7 | **97**.5 | 79.0 |
| 2 | Decision tree | 69 | 60.3 | 40.3 | 39.3 | 41.4 | 69.4 | 56.0 |
| 3 | XGBoost | 69 | 77.7 | 63.6 | 67.3 | 60.3 | 86.0 | 81.0 |
| 4 | Support Vector Machine - RBF | 69 | 73.7 | 48.4 | 66.7 | 37.9 | 90.9 | 77.0 |
| 5 | Linear support vector machine | 69 | 77.7 | 66.1 | 65.0 | 67.2 | 82.6 | 81.0 |
| 6 | Gaussian Process | 69 | 70.9 | 42.2 | 59.4 | 32.8 | 89.3 | 68.0 |
| 7 | Simple Bayes | 69 | 41.3 | 47.2 | 33.3 | **81.0** | 22.3 | 69.0 |
| 8 | K Nearest neighbor | 69 | 69.8 | 37.2 | 57.1 | 27.6 | 90.1 | 64.0 |
| 9 | Logistic regression | 69 | **79.9** | **67.9** | **70.4** | 65.5 | 86.8 | **81.0** |
| 10 | Neural networks | 69 | 74.9 | 49.4 | 71.0 | 37.9 | 93.0 | 65.2 |
|  | Reduced LASSO model | | |  |  |  |  |  |
| 1 | Random Forest | 19 | 73.2 | 46.7 | 65.6 | 36.2 | 90.9 | 73.0 |
| 2 | Decision tree | 19 | 68.7 | 50.9 | 51.8 | 50.0 | 77.7 | 64.0 |
| 3 | XGBoost | 19 | 69.3 | 57.4 | 52.1 | 63.8 | 71.9 | 76.0 |
| 4 | Support Vector Machine - RBF | 19 | 70.4 | 34.6 | 60.9 | 24.1 | 92.6 | 74.0 |
| 5 | Linear support vector machine | 19 | 77.7 | 67.2 | 64.1 | 70.7 | 81.0 | 82.0 |
| 6 | Gaussian Process | 19 | 73.7 | 44.7 | **70.4** | 32.8 | 93.4 | 74.0 |
| 7 | Simple Bayes | 19 | 69.3 | 48.6 | 53.1 | 44.8 | 81.0 | 74.0 |
| 8 | K Nearest neighbor | 19 | 70.9 | 40.9 | 60.0 | 31.0 | 90.1 | 64.0 |
| 9 | Logistic regression | 19 | **80.4** | **71.1** | 68.3 | **74.1** | 83.5 | 82.0 |
| 10 | Neural networks | 19 | 73.2 | 52.9 | 61.4 | 46.5 | 100 | 66.2 |
|  | Reduced Random Forest Model | | | |  |  |  |  |
| 1 | Random Forest | 20 | 72.1 | 45.7 | 61.8 | 36.2 | 89.3 | 76.0 |
| 2 | Decision tree | 20 | 62.6 | 44.6 | 42.9 | 46.6 | 70.2 | 59.0 |
| 3 | XGBoost | 20 | 72.1 | 57.6 | 56.7 | 58.6 | 78.5 | 77.0 |
| 4 | Support Vector Machine - RBF | 20 | 58.1 | 57.1 | 42.7 | **86.2** | 44.6 | 76.0 |
| 5 | Linear support vector machine | 20 | 73.7 | 64.1 | 57.5 | 72.4 | 74.4 | 80.0 |
| 6 | Gaussian Process | 20 | 71.5 | 33.8 | **68.4** | 22.4 | 95.0 | 69.0 |
| 7 | Simple Bayes | 20 | 69.8 | 54.2 | 53.3 | 55.2 | 76.9 | 74.0 |
| 8 | K Nearest neighbor | 20 | 70.9 | 39.5 | 60.7 | 29.3 | 90.9 | 70.0 |
| 9 | Logistic regression | 20 | **75.4** | **65.1** | 60.3 | 70.7 | 77.7 | **80.0** |
| 10 | Neural networks | 20 | 72.1 | 43.2 | 63.3 | 32.7 | 86.0 | 61.8 |
| LASSO: Least Absolute Shrinkage and Selection Operator, SINBAD: Site Ischemia Neuropathy Bacterial Infection and Depth RBF: Radial Basis Function | | | | | | | | |

**Performance of the Infectious Diseases Society of America (IDSA) Wound Classification System in Predicting Non-Healing Outcomes**

Table S8 presents the performance of the Infectious Diseases Society of America (IDSA) wound classification system in predicting non-healing outcomes over a six-month follow-up period. Logistic regression emerged as the best-performing algorithm in the full model, the reduced LASSO model, and the reduced Random Forest model.

When comparing models based on their best-performing algorithms, the reduced Random Forest model demonstrated superior performance. It achieved a higher F1 score (66.1) compared to the full model (58.9) and the reduced LASSO model (64.5). Additionally, its sensitivity was higher (69.0%) than both the full model (56.9%) and the LASSO model (67.2%). Therefore, logistic regression in the reduced Random Forest model was selected for comparing the IDSA wound classification system with other wound classification systems.

Table S8: Performance of the IDSA Wound Classification System in Predicting unhealing Outcome Over a Six-Month Evaluation Period

|  | Machine learning algorithm | number of Variable | Accuracy | F1 score | precision | Sensitivity | specificity | AUC |
| --- | --- | --- | --- | --- | --- | --- | --- | --- |
|  | Full model | | |  |  |  |  |  |
| 1 | Random Forest | 64 | 73.2 | 40.0 | 72.7 | 27.6 | 95.0 | 78.0 |
| 2 | Decision tree | 64 | 61.5 | 31.7 | 37.2 | 27.6 | 77.7 | 54.0 |
| 3 | XGBoost | 64 | 72.1 | 56.1 | 57.1 | 55.2 | 80.2 | 77.0 |
| 4 | Support Vector Machine - RBF | 64 | 72.1 | 51.0 | 59.1 | 44.8 | 85.1 | 76.0 |
| 5 | Linear support vector machine | 64 | 73.2 | 58.6 | 58.6 | 58.6 | 80.2 | 77.0 |
| 6 | Gaussian Process | 64 | 71.5 | 42.7 | 61.3 | 32.8 | 90.1 | 66.0 |
| 7 | Simple Bayes | 64 | 39.7 | 48.1 | 33.3 | **86.2** | 17.4 | 69.0 |
| 8 | K Nearest neighbor | 64 | 71.5 | 44.0 | 60.6 | 34.5 | 89.3 | 64.0 |
| 9 | Logistic regression | 64 | **74.3** | **58.9** | 61.1 | 56.9 | 82.6 | **78.0** |
| 10 | Neural networks | 64 | 74.3 | 43.9 | **75.0** | 31.0 | 95.0 | 63.0 |
|  | Reduced LASSO model | | |  |  |  |  |  |
| 1 | Random Forest | 16 | 72.1 | 50.0 | 59.5 | 43.1 | 86.0 | 74.0 |
| 2 | Decision tree | 16 | 64.8 | 45.2 | 45.6 | 44.8 | 74.4 | 60.0 |
| 3 | XGBoost | 16 | 72.6 | 58.1 | 57.6 | 58.6 | 79.3 | 76.0 |
| 4 | Support Vector Machine - RBF | 16 | 68.2 | 38.7 | 51.4 | 31.0 | 86.0 | 74.0 |
| 5 | Linear support vector machine | 16 | 70.9 | 62.3 | 53.8 | **74.1** | 69.4 | **78.0** |
| 6 | Gaussian Process | 16 | 69.3 | 36.8 | 55.2 | 27.6 | 89.3 | 72.0 |
| 7 | Simple Bayes | 16 | 70.9 | 49.0 | 56.8 | 43.1 | 84.3 | 71.0 |
| 8 | K Nearest neighbor | 16 | 71.5 | 41.4 | 62.1 | 31.0 | 90.9 | 65.0 |
| 9 | Logistic regression | 16 | **76.0** | **64.5** | 61.9 | 67.2 | 80.2 | 77.0 |
| 10 | Neural networks | 16 | 74.3 | 52.1 | **65.8** | 43.1 | 100 | 66.2 |
|  | Reduced Random Forest Model | | |  |  |  |  |  |
| 1 | Random Forest | 20 | 71.5 | 42.7 | 61.3 | 32.8 | 90.1 | 77.0 |
| 2 | Decision tree | 20 | 67.0 | 50.4 | 49.2 | 51.7 | 74.4 | 62.0 |
| 3 | XGBoost | 20 | 72.1 | 56.1 | 57.1 | 55.2 | 80.2 | **78.0** |
| 4 | Support Vector Machine - RBF | 20 | 73.7 | 58.4 | 60.0 | 56.9 | 81.8 | 75.0 |
| 5 | Linear support vector machine | 20 | 77.1 | 65.5 | 63.9 | 67.2 | 81.8 | 76.0 |
| 6 | Gaussian Process | 20 | 74.3 | 45.2 | 73.1 | 32.8 | 94.2 | 66.0 |
| 7 | Simple Bayes | 20 | 72.6 | 51.5 | 60.5 | 44.8 | 86.0 | 74.0 |
| 8 | K Nearest neighbor | 20 | 73.7 | 46.0 | 69.0 | 34.5 | 92.6 | 74.0 |
| 9 | Logistic regression | 20 | 77.1 | **66.1** | 63.5 | **69.0** | 81.0 | 76.0 |
| 10 | Neural networks | 20 | **74.9** | 41.5 | **84.2** | 27.6 | 95.0 | 62.5 |
| LASSO: Least Absolute Shrinkage and Selection Operator, IDSA: Infectious Diseases Society of America, RBF: Radial Basis Function | | | | | | | | |

**Performance of the PEDIS Wound Classification System in Predicting Non-Healing Outcome**

Table S9 presents the performance of the PEDIS wound classification system in predicting non-healing outcomes over a six-month follow-up period. The linear support vector machine algorithm performed best in both the full model and the reduced Random Forest model, while logistic regression was the best-performing algorithm in the reduced LASSO model.

When comparing the models based on their best-performing algorithms, the reduced LASSO and Random Forest models showed similar performance across most indicators, except for the area under the curve (AUC), which was higher in the LASSO model. Specifically, the reduced LASSO model demonstrated higher F1 score (68.3 vs. 67.9), sensitivity (72.4% vs. 65.5%), and AUC (83.0 vs. 82.0) than the full model, while also using fewer variables. Therefore, logistic regression in the reduced LASSO model was selected to compare the PEDIS classification system with other wound classification systems.

Table S9: Performance of the PEDIS Wound Classification System in Predicting unhealing Outcome Over a Six-Month Evaluation Period

|  | Machine learning algorithm | number of Variable | Accuracy | F1 score | precision | Sensitivity | specificity | AUC |
| --- | --- | --- | --- | --- | --- | --- | --- | --- |
|  | Full model | | | |  |  |  |  |
| 1 | Random Forest | 68 | 74.3 | 51.1 | 66.7 | 41.4 | 90.1 | 79.0 |
| 2 | Decision tree | 68 | 59.8 | 32.1 | 35.4 | 29.3 | 74.4 | 52.0 |
| 3 | XGBoost | 68 | 76.5 | 63.2 | 64.3 | 62.1 | 83.5 | 81.0 |
| 4 | Support Vector Machine - RBF | 68 | 76.5 | 63.8 | 63.8 | 63.8 | 82.6 | 79.0 |
| 5 | Linear support vector machine | 68 | **79.9** | **67.9** | **70.4** | 65.5 | 86.8 | 82.0 |
| 6 | Gaussian Process | 68 | 70.4 | 48.5 | 55.6 | 43.1 | 83.5 | 69.0 |
| 7 | Simple Bayes | 68 | 41.9 | 47.5 | 33.6 | **81.0** | 23.1 | 69.0 |
| 8 | K Nearest neighbor | 68 | 69.3 | 38.2 | 54.8 | 29.3 | 88.4 | 66.0 |
| 9 | Logistic regression | 68 | 78.8 | 66.1 | 68.5 | 63.8 | 86.0 | **84.0** |
| 10 | Neural networks | 68 | 69.8 | 37.2 | 57.1 | 27.6 | 90.0 | 58.8 |
|  | Reduced LASSO model | | | |  |  |  |  |
| 1 | Random Forest | 18 | 73.7 | 52.5 | 63.4 | 44.8 | 87.6 | 74.0 |
| 2 | Decision tree | 18 | 63.1 | 42.1 | 42.9 | 41.4 | 73.6 | 58.0 |
| 3 | XGBoost | 18 | 75.4 | 62.7 | 61.7 | 63.8 | 81.0 | 81.0 |
| 4 | Support Vector Machine - RBF | 18 | 77.7 | 62.3 | 68.8 | 56.9 | 87.6 | 77.0 |
| 5 | Linear support vector machine | 18 | 71.5 | 61.7 | 54.7 | 70.7 | 71.9 | 83.0 |
| 6 | Gaussian Process | 18 | 74.3 | 51.1 | 66.7 | 41.4 | 90.1 | 75.0 |
| 7 | Simple Bayes | 18 | **79.9** | 66.0 | **72.9** | 60.3 | 89.3 | 79.0 |
| 8 | K Nearest neighbor | 18 | 65.4 | 31.1 | 43.8 | 24.1 | 85.1 | 62.0 |
| 9 | Logistic regression | 18 | 78.2 | **68.3** | 64.6 | **72.4** | 81.0 | **83.0** |
| 10 | Neural networks | 18 | 77.6 | 53.5 | 82.1 | 39.6 | 96.0 | 67.8 |
|  | Reduced Random Forest Model | | | |  |  |  |  |
| 1 | Random Forest | 20 | 74.3 | 47.7 | **70.0** | 36.2 | 92.6 | 75.0 |
| 2 | Decision tree | 20 | 60.9 | 36.4 | 38.5 | 34.5 | 73.6 | 54.0 |
| 3 | XGBoost | 20 | 74.3 | 60.3 | 60.3 | 60.3 | 81.0 | 78.0 |
| 4 | Support Vector Machine - RBF | 20 | 75.4 | 62.7 | 61.7 | 63.8 | 81.0 | 78.0 |
| 5 | Linear support vector machine | 20 | **78.2** | **68.3** | 64.6 | 72.4 | 81.0 | **81.0** |
| 6 | Gaussian Process | 20 | 72.6 | 42.4 | 66.7 | 31.0 | 92.6 | 72.0 |
| 7 | Simple Bayes | 20 | **78.2** | 66.1 | 66.7 | 65.5 | 84.3 | 79.0 |
| 8 | K Nearest neighbor | 20 | 71.5 | 42.7 | 61.3 | 32.8 | 90.1 | 74.0 |
| 9 | Logistic regression | 20 | 77.7 | **68.3** | 63.2 | **74.1** | 79.3 | 80.0 |
| 10 | Neural networks | 20 | 67.6 | 32.6 | 50.0 | 24.1 | 88.0 | 56.3 |
| LASSO: Least Absolute Shrinkage and Selection Operator, PEDIS: perfusion extent depth/tissue loss infection and sensation , RBF: Radial Basis Function | | | | | | | | |

**Performance of the DiaFORA Wound Classification System in Predicting Non-Healing Outcome**

Table S10 presents the performance of the DiaFORA wound classification system in predicting non-healing outcomes over a six-month follow-up period. The linear support vector machine algorithm showed the best performance in both the full model and the reduced LASSO model, while logistic regression performed best in the reduced Random Forest model.

When comparing the models based on their best-performing algorithms, the full model demonstrated superior performance in terms of accuracy, F1 score, precision, and specificity compared to the reduced LASSO and Random Forest models. However, the LASSO model achieved higher sensitivity than both the full and Random Forest models. Given the reduced number of variables and improved sensitivity, the linear support vector machine algorithm in the reduced LASSO model was selected for comparing the DiaFORA classification system with other wound classification systems.

Table S10: Performance of the DIaFORA Wound Classification System in Predicting unhealing Outcome Over a Six-Month Evaluation Period

|  | Machine learning algorithm | number of Variable | Accuracy | F1 score | precision | Sensitivity | specificity | AUC |
| --- | --- | --- | --- | --- | --- | --- | --- | --- |
|  | Full model | | |  |  |  |  |  |
| 1 | Random Forest | 70 | 73.2 | 42.9 | 69.2 | 31.0 | 93.4 | 81.0 |
| 2 | Decision tree | 70 | 68.2 | 48.6 | 50.9 | 46.6 | 78.5 | 64.0 |
| 3 | XGBoost | 70 | 77.7 | 63.0 | 68.0 | **58.6** | 86.8 | 82.0 |
| 4 | Support Vector Machine - RBF | 70 | 70.4 | 43.0 | 57.1 | 34.5 | 87.6 | 78.0 |
| 5 | Linear support vector machine | 70 | **80.4** | **65.3** | **76.7** | 56.9 | **91.7** | **82.0** |
| 6 | Gaussian Process | 70 | 68.7 | 37.8 | 53.1 | 29.3 | 87.6 | 65.0 |
| 7 | Simple Bayes | 70 | 41.3 | 47.2 | 33.3 | 81.0 | 22.3 | 70.0 |
| 8 | K Nearest neighbor | 70 | 69.3 | 36.8 | 55.2 | 27.6 | 89.3 | 62.0 |
| 9 | Logistic regression | 70 | 77.7 | 58.3 | 73.7 | 48.3 | 91.7 | 82.0 |
| 10 | Neural networks | 70 | 70.4 | 52.2 | 54.7 | 50.0 | 80.0 | 65.1 |
|  | Reduced LASSO model | | |  |  |  |  |  |
| 1 | Random Forest | 23 | 72.1 | 47.9 | 60.5 | 39.7 | 87.6 | 76.0 |
| 2 | Decision tree | 23 | 64.2 | 47.5 | 45.3 | 50.0 | 71.1 | 61.0 |
| 3 | XGBoost | 23 | **76.0** | **63.2** | 62.7 | 63.8 | 81.8 | 80.0 |
| 4 | Support Vector Machine - RBF | 23 | 70.4 | 34.6 | 60.9 | 24.1 | 92.6 | 74.0 |
| 5 | Linear support vector machine | 23 | 73.7 | **63.6** | 57.7 | **70.7** | 75.2 | **82.0** |
| 6 | Gaussian Process | 23 | 69.8 | 28.9 | 61.1 | 19.0 | 94.2 | 73.0 |
| 7 | Simple Bayes | 23 | 73.2 | 54.7 | 60.4 | 50.0 | 84.3 | 76.0 |
| 8 | K Nearest neighbor | 23 | 72.6 | 34.7 | **76.5** | 22.4 | 96.7 | 71.0 |
| 9 | Logistic regression | 23 | 73.7 | 58.4 | 60.0 | 56.9 | 81.8 | **82.0** |
| 10 | Neural networks | 23 | 69.8 | 32.5 | 59.1 | 22.4 | 100 | 57.5 |
|  | Reduced Random Forest Model | | |  |  |  |  |  |
| 1 | Random Forest | 20 | 69.8 | 38.6 | 56.7 | 29.3 | 89.3 | 76.0 |
| 2 | Decision tree | 20 | 69.3 | 51.3 | 52.7 | 50.0 | 78.5 | 64.0 |
| 3 | XGBoost | 20 | 73.2 | 56.4 | 59.6 | 53.4 | 82.6 | 77.0 |
| 4 | Support Vector Machine - RBF | 20 | 71.5 | 62.8 | 54.4 | **74.1** | 70.2 | 78.0 |
| 5 | Linear support vector machine | 20 | 73.7 | 62.4 | 58.2 | 67.2 | 76.9 | 80.0 |
| 6 | Gaussian Process | 20 | 70.4 | 25.4 | **69.2** | 15.5 | 96.7 | 68.0 |
| 7 | Simple Bayes | 20 | 76.5 | 61.1 | 66.0 | 56.9 | 86.0 | 74.0 |
| 8 | K Nearest neighbor | 20 | 68.2 | 34.5 | 51.7 | 25.9 | 88.4 | 65.0 |
| 9 | Logistic regression | 20 | **76.5** | **63.2** | 64.3 | 62.1 | 83.5 | **81.0** |
| 10 | Neural networks | 20 | 64.2 | 25.6 | 39.3 | 19.0 | 93.0 | 52.4 |
| LASSO: Least Absolute Shrinkage and Selection Operator, DiaFORA: Diabetic Foot Risk Assessment PEDIS: perfusion extent depth/tissue loss infection and sensation , RBF: Radial Basis Function | | | | | | | | |
